# Clinical and genomic characterization of chemoradiation-resistant HPV-positive oropharyngeal squamous cell carcinoma

**DOI:** 10.3389/fonc.2024.1336577

**Published:** 2024-03-05

**Authors:** Theresa Guo, Fernando Zamuner, Stephanie Ting, Liam Chen, Lisa Rooper, Pablo Tamayo, Carole Fakhry, Daria Gaykalova, Ranee Mehra

**Affiliations:** ^1^ Department of Otolaryngology, Moores Cancer Center, University of California, San Diego, San Diego, CA, United States; ^2^ Department of Otolaryngology-Head and Neck Surgery, Johns Hopkins University, Baltimore, MD, United States; ^3^ Department of Medicine, Division of Hematology-Oncology, University of California, San Diego, San Diego, CA, United States; ^4^ Division of Neuropathology, Department of Pathology, University of Minnesota Medical School, Minneapolis, MN, United States; ^5^ Department of Pathology, Johns Hopkins Hospital, Baltimore, MD, United States; ^6^ Department of Otorhinolaryngology-Head and Neck Surgery, University of Maryland School of Medicine, Baltimore, MD, United States; ^7^ Marlene and Stewart Greenebaum Comprehensive Cancer Center, University of Maryland School of Medicine, Baltimore, MD, United States; ^8^ Institute for Genome Sciences, University of Maryland School of Medicine, Baltimore, MD, United States; ^9^ Department of Oncology, Sidney Kimmel Comprehensive Cancer Center, Johns Hopkins University, Baltimore, MD, United States

**Keywords:** HPV, oropharyngeal squamous cell carcinoma, platinum resistance, treatment resistance, persistent disease, genomics

## Abstract

**Introduction:**

Most patients with HPV-positive oropharyngeal squamous cell carcinoma (OPSCC) have an excellent response to chemoradiation, and trials are now investigating de-escalated treatment. However, up to 25% of patients with HPV-positive OPSCC will experience recurrence, and up to 5% will even progress through primary treatment. Currently, there are no molecular markers to identify patients with poor prognosis who would be harmed by de-escalation. Herein we report the clinical and genomic characteristics of persistent HPV-positive OPSCC after definitive platinum-based chemoradiation therapy.

**Methods:**

Patients with HPV-positive OPSCC treated with curative intent platinum-based chemoradiation between 2007 and 2017 at two institutions and with a persistent locoregional disease were included. We evaluated clinical characteristics, including smoking status, age, stage, treatment, and overall survival. A subset of five patients had tissue available for targeted exome DNA sequencing and RNA sequencing. Genomic analysis was compared to a previously published cohort of 47 treatment-responsive HPV+ OPSCC tumors after batch correction. Mutational landscape, pathway activation, and OncoGPS tumor states were employed to characterize these tumors.

**Results:**

Ten patients met the inclusion criteria. The tumor and nodal stages ranged from T1 to T4 and N1 to N2 by AJCC 8th edition staging. All patients were p16-positive by immunohistochemistry, and eight with available *in situ* hybridization were confirmed to be HPV-positive. The 1-year overall survival from the time of diagnosis was 57%, and the 2-year overall survival was 17%. *TP53* mutations were present in three of five (60%) persistent tumors compared to 2% (one of 47) of treatment-responsive HPV-positive tumors (*p* = 0.008). Other genes with recurrent mutations in persistent HPV-positive OPSCC tumors were *NF1*, *KMT2D*, *PIK3C2B*, and *TFGBR2*. Compared to treatment-responsive HPV-positive tumors, persistent tumors demonstrated activation of DNA Repair and p53, EMT, MYC, SRC, and TGF-beta signaling pathways, with post-treatment samples demonstrating significant activation of the PI3K-EMT-Stem pathways compared to pretreatment samples.

**Conclusion:**

Chemoradiation-resistant HPV-positive OPSCC occurs infrequently but portends a poor prognosis. These tumors demonstrate higher rates of p53 mutation and activation of MYC, SRC, and TGF-beta pathways. A comparison of tumors before and after treatment demonstrates PI3K-EMT-Stem pathways post-treatment in HPV-positive tumors with persistent disease after platinum-based chemoradiation.

## Introduction

In recent years, the prevalence of HPV-positive oropharyngeal squamous cell carcinoma (OPSCC) has increased significantly, and the incidence of OPSCC has now surpassed that of cervical cancer in both the US and the UK (1, 2). Currently, over 70% of all oropharynx tumors are HPV-positive (1). In addition, it is now recognized that a HPV-positive tumor status is associated with a good prognosis and improved response to chemoradiation (3, 4). Accordingly, the new AJCC staging system has incorporated HPV tumor status, and a HPV-positive status significantly downstages a tumor (4). Several trials are actively investigating the de-escalation of therapy for HPV-positive OPSCC, given significantly improved overall prognosis (5, 6).

However, in contrast to this paradigm, up to 25% of patients with HPV-positive OPSCC will experience recurrence, and up to 5% will demonstrate a lack of response to primary treatment, representing a persistent disease (3, 4). These patients have tumors that do not respond to standard chemoradiation and are without a disease-free interval. By the first post-treatment evaluation at 12 weeks, they demonstrate a persistent or progressive disease. This atypical aggressive clinical behavior is distinct in patients who experience a recurrent disease with a disease-free interval, often years after the definitive treatment. Similar to other patients with a HPV-positive disease, some of these disease-persistent patients may not even have a history of smoking or alcohol use and may be relatively young. In short, the biology of their disease is inconsistent with what we understand about HPV-related tumors. Those with treatment resistance are rare but portend dire prognosis and have not been well characterized. There are currently no biomarkers to identify patients who will not respond to treatment or would potentially be harmed by de-escalation.

Therefore, the goal of this study is to characterize HPV-positive tumors that demonstrate persistence and a lack of treatment response to standard platinum-based chemoradiation treatment through targeted DNA sequencing and RNA sequencing. These tumors were then compared to previously sequenced cohorts: HPV-positive tumors with good prognosis (no progression with 3 years follow-up) and from The Caner Genome Atlas (TCGA). A comparison of the mutational landscape and gene expression profiles for these cohorts will seek to identify potential biomarkers and key oncogenic pathways for HPV-positive tumors with poor prognosis. While these cases are rare, the ability to identify patients with this aggressive disease would inform which patients are not candidates for de-escalation of therapy and provide insight into the biomarkers of poor prognosis in a HPV-positive disease.

## Methods

### Patient cohort

This study was approved as a part of IRB protocol NA_00036235 through Johns Hopkins Medicine IRB. Patients were included if they completed platinum-based chemoradiation with curative intent for HPV-positive oropharyngeal squamous cell carcinoma between 2007 and 2017 at two institutions, Johns Hopkins Hospital and Greater Baltimore Medical Center, and demonstrated a locoregional persistent disease or progression after treatment. Clinical characteristics, including smoking status, age, stage, primary treatment, and overall survival, were abstracted from the medical record. Patients were excluded if they did not complete the standard-of-care curative intent treatment, were not treated with concurrent platinum-based treatment, had out-of-field or metastatic recurrence without a locoregional disease, or were lost to follow-up.

### Patient samples

Patient samples were obtained from archived paraffin-embedded tissue from a biopsy or surgical specimens. Only a subset of patients had available archival biopsy or resection tissue for DNA/RNA extraction and subsequent sequencing. Patients with original biopsies performed outside of our institution did not have tissue available for additional sequencing analysis. The diagnosis of squamous cell carcinoma was confirmed by pathology, and HPV tumor status was evaluated with p16 immunohistochemistry with >70% staining. In eight out of 10 patients, HPV *in situ* hybridization was also available to confirm HPV positivity. Pre-treatment specimens and additional post-treatment samples of a persistent disease were also obtained when available. Two patients had paired pre- and post-treatment tissue samples that were available for analysis. Additional comparisons were performed with a previously published HPV-positive cohort with 47 tumors and 25 normal samples (7). Data from the Cancer Genome Atlas (TCGA) was utilized for a comparative mutational analysis. A total of 497 head and neck squamous cell carcinoma (HNSCC) tumors from The Cancer Genome Atlas (TCGA) were also utilized for tumor state analysis, including 44 HPV-positive oropharyngeal tumors (8).

### Sample preparation and sequencing

Next-generation sequencing was performed as part of routine clinical care for three patients. Targeted exome data was obtained from FoundationOne (Cambridge, MA, USA) for two patients and PGDx (Personal Genome Diagnostics, Baltimore, MD, USA) for one patient. These analyses included the targeted sequencing of 125–315 cancer-related genes. Remaining available tissue was obtained in these patients from unstained paraffin-embedded slides from biopsy or surgical specimens. The tissue slides were reviewed by a pathologist, and tumor tissue was defined on each slide to enrich the tumor collection on tumor slides. Matched adjacent normal tissue from cancer patients was obtained from two patients to include for normal comparison and for somatic mutation analysis. Five slides per patient sample were utilized for tissue collection. The tissue from slides was then collected, and combined DNA and RNA extraction was performed using the AllPrep DNA/RNA extraction kit (Qiagen). All tumor samples were processed in a single batch with two matched normal controls (adjacent normal tissue from two cohort patients with persistent disease) and tissue from three previously sequenced tumors in patients with no evidence of a recurrent disease to be used for batch correction with the previously published cohort (7). Due to the small size of the biopsy tissue, one patient with targeted DNA exome sequencing did not have any remaining tissue available for RNA extraction and sequencing.

RNA sequencing and DNA sequencing were performed at the Johns Hopkins Genetic Resource Core Facility (GRCF). Targeted exome sequencing was performed on two patients with paired normal tissue to allow for mutation calling on a panel of 434 cancer-associated genes in the curated solid tumor panel (**Supplementary Table S1**). Sequencing was conducted using NovaSeq system (Illumina) as previously described (9). Library preparation was performed using SureSelect-XT Target Enrichment System (Agilent Technologies) with a curated cancer-related solid tumor gene panel. The read depth ranged from 100 to 500×. Reads were aligned to the human genome (GrCh37/hg19) using Burrows–Wheeler alignment. Variant calling was performed using in-house variant caller algorithm (MDLVC v5.0) and HaplotypeCaller (Genome Analysis Tool Kit 3.3). These were reviewed using Integrated Genomics Viewer (Broad Institute). Variants with strand bias, low coverage (<300), or consistent with artifact after review were removed. As mentioned above, the remaining exome sequencing was obtained from clinical genomic testing. Mutational profiles of each sample are available in **Supplementary Table S2**.

For RNA sequencing, samples were required to achieve an RNA Integrity Number (RIN) of at least 7.0. Barcoded and stranded libraries from ribosomal RNA depleted RNA were prepared using Takara SMARTer Total RNA-Seq Kit v2. Sequencing was performed using Illumina NovaSeq 6000 platform. Alignment was performed to GRCH37/hg19 genome assembly using the salmon aligner (10), and gene expression profiles were extracted. A similar pipeline was used to realign 47 HPV+ OPSCC tumors and 25 oropharyngeal mucosae from normal controls from our previously published patient cohort (7), including four patients with a recurrent disease at a median follow-up of 31 months to allow for batch correction and gene expression comparison.

### Gene expression analysis

Batch correction and gene expression analysis were performed using DESeq in R (11), version 4.0.4. Batch correction then allowed integrated gene expression analysis between persistent tumors and the previously published cohort (7). Batch corrected gene abundances are included in **Supplementary Table S3**. Gene Set Enrichment Analysis (GSEA) was performed to compare differentially expressed genes between cohorts (12). Pathway activation was scored utilizing the Denoising Algorithm based on Relevance network Topology (DART) (13) and published pathway activation signatures. PD1 (PDCD1) and PDL1 (CD274) expressions were directly compared after batch correction. Deconvolution of the estimated immune infiltrates was evaluated using xCell (14). The estimated cell type enrichment scores were compared after batch correction, and multiple testing correction was performed using the Benjamini–Hochberg method.

### OncoGPS tumor states

Additional genomic modeling was performed by projecting gene expression profiles onto an archetype map of head and neck squamous cell carcinoma using Onco-GPS methods (15). Briefly, the characteristic cancer pathways characteristic of head and neck squamous cell carcinoma were selected *a priori*: NfkB-SRC-JUN pathway, TCA Cycle, DNA Repair-MYC-E2F, PI3K-EMT-Stem, WNT-BCAT-AKT, NFkB-IRF-KLF5, EGFR-p63, EMT-ZEB1, and NRF2-PPP pathways (**Supplementary Figure S1**). Utilizing projections of these gene set pathways, nine tumor states were defined within head and neck squamous cell carcinoma by utilizing 497 head and neck squamous cell carcinoma tumors from The Cancer Genome Atlas (TCGA). TCGA tumors were then categorized into these nine states based on master transcriptional components, and tumor characteristics were described within each state including tumor subsite, HPV status, *TP53* mutation status, and smoking history. Lastly, non-recurrent and persistent HPV-positive tumors were projected and defined onto these tumor states based on the pathway activation of nine master transcriptional components, and pre- and post-treatment tumor states were compared for patients for which biopsy samples were available before and after chemoradiation.

### Survival analysis

A survival analysis was performed using the “survival” package from CRAN, version 3.3-1, using R version 4.0.4. The survival data was compared between groups using Kaplan–Meier and log rank statistics. Similar methods were utilized for the evaluation of our clinical cohort patients and TCGA patients. Additional survival analyses were performed to compare overall survival based on mutational status.

## Results

### Clinical characteristics and outcomes

Of 18 patients identified with persistent or progressive HPV-positive oropharyngeal squamous cell carcinoma, 10 patients who completed curative intent chemoradiation with treatment to 70 Gy and concurrent platinum-based chemotherapy (cisplatin or carboplatin) met the inclusion criteria (**Table 1**). Patients were excluded if they received cetixumab (*n* = 1), did not complete recommended chemotherapy or radiation course due to side effects (*n* = 2), only had metastatic recurrence without locoregional persistence (*n* = 3), had out-of-field recurrence (*n* = 1), or were lost to follow up (*n* = 1). All patients were male, with a median age of 58 years. The patients included 30% of never smokers and 40% of former smokers. The tumor and nodal stages ranged from T1 to T4 and N1 to N2 by AJCC 8th edition staging. All patients were p16-positive by immunohistochemistry, and eight patients with available HPV *in situ* hybridization (ISH) were confirmed to be HPV-positive. All patients were treated with definitive chemoradiation with evidence of locoregional persistence after treatment.

**Table 1:** Patient cohort.

		Smoker	AJCC 8th edition staging	Primary treatment	Response and treatment	Follow-up, months	Alive at FU	TMB	p53	RNA		DNA	
										Pre	Post	Pre	Post
1	59, M	Never	T1N1M0	CRT with cisplatin	LR persistence, new spinal mets: hospice care	5.65	N	9	WT	X		X	
2	43, M	Never	T1N1M0	CRT with cisplatin	LR persistence: salvage surgery	13.94	N	5	WT		X		X
3^a^	55, M	Former (15 pky)	T3N2M0	CRT with cisplatin	LR persistence: hospice care	4.66	N	36	D228H	X	X	X	
4	87, M	Former (7.5 pky)	T2N2M0	CRT with carboplatin	LR persistence: immunotherapy, carboplatin	8.12	Y	–	R123X			X	
5^a^	49, M	Current (30 pky)	T4N2M0	CRT with carboplatin/paclitaxel	LR persistence, new liver mets: immunotherapy trial	9.73	N	2	R181C	X	X		X
6	75, M	Former (10 pky)	T3N1M0	CRT with cisplatin	LR persistence, new lung mets: carbo/taxol	9.47	N						
7	58, M	Never	T2N1M0	CRT with cisplatin	LR persistence: salvage surgery	15.85	Y						
8	54, M	Former (15 pky)	T2N1M0	CRT with cisplatin	LR persistence: salvage surgery	12.39	N						
9	58, M	Former (27 pky)	T4N2M0	CRT with cisplatin, cetuximab, and paclitaxel	LR persistence, new lung mets: cis/cetux/paclitaxel	20.25	N						
10	64, M	Current (45 pky)	T3N2M0	CRT with cisplatin	LR persistance: salvage surgery	27.98	N						

pky, pack years; CRT, chemoradiation; LR, locoregional; FU, follow-up; TMB, tumor mutation burden in mut/Mb.

a Patients with paired pre- and post-treatment biopsies.

**Figure 1:**
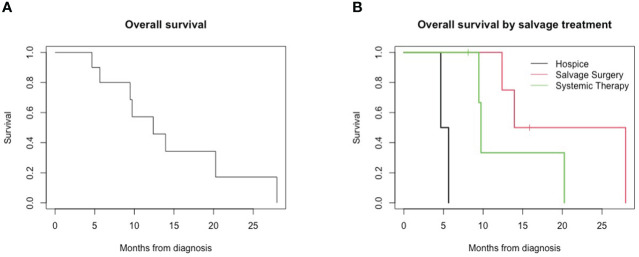
Overall survival for patients with persistent HPV-positive oropharyngeal squamous cell carcinoma. **(A)** Overall survival of all patients. **(B)** Overall survival by salvage treatment.

All patients demonstrated a persistent locoregional disease after completion of primary therapy, assessed within 4 months of treatment by the first post-treatment imaging study or sooner if clinically evident persistence was present. All patients demonstrated no disease-free interval. In addition, four patients (40%) developed new distant metastases upon follow-up imaging at the time of first post-treatment evaluation at 4 months, including lung, bone, and liver metastases. Median overall survival was 12.4 months. The 1-year overall survival from the time of diagnosis was 57%, and the 2-year survival was 17% (**Figure 1**). Median survival in patients with locoregional persistence was 13.9 months compared to 9.6 months in those with concurrent metastatic disease.

Two patients were enrolled in hospice care following the diagnosis of a persistent disease, and survival for both patients was less than 6 months from the initial oncologic diagnosis. Four patients underwent salvage surgery for the persistent locoregional disease, and four patients underwent treatment with systemic therapy with chemotherapy and/or immunotherapy. Overall survival in this small cohort differed for patients based on salvage treatment received; patients receiving hospice (median, 5.16 months) had lower overall survival compared to those receiving systemic therapy (9.73 months) and salvage surgery (20.96 months) (**Figure 1**, *p* = 0.002).

### Mutational analysis

TCGA showed that HPV-negative tumors have a high rate of *TP53* mutation, while these mutations are quite rare in HPV-positive tumors. DNA sequencing demonstrated that *TP53* mutations were present in three of five (60%) persistent HPV-positive tumors compared to 2% of treatment-responsive HPV-positive tumors from the previously published cohort (*p* = 0.008). This is consistent with the *TP53* mutation rate in TCGA of 2.2% of 45 HPV-positive tumors. The identified *TP53* mutations in persistent tumors were D228H, R123X, and R181C. These mutations were all within the DNA binding domain of p53, but they were not identified in known mutational hotspots (16).

Within the entire TCGA HNSCC cohort (*n* = 506 with available mutation data) including both HPV-positive and HPV-negative cohorts, p53 mutations were identified in 71% of patients. The presence of *TP53* mutation was associated with worse overall survival (median OS, 45.8 vs. 65.8 months, *p* = 0.009). This was even more pronounced among HPV-positive TCGA patients (median OS, 12.2 vs. 68.4 months, *p* < 0.001; p53 mutation rate 2.2%, **Supplementary Figure S2**). As has been previously published (17), p53 mutations were associated with smoking; mutations were identified in 79% of current smokers compared to 65% of non-smokers (*p* = 0.009). HPV-positive patients within control cohorts that harbored p53 mutations included both a current smoker (TCGA) and non-smoker (previously published cohort) (18). The mutation in *TP53* was associated with worse overall survival in smokers (*p* = 0.01) but not in never smokers (*p* = 0.3).

Other genes harboring mutations among 40% (*n* = 2) of persistent HPV-positive OPSCC tumors were *NF1*, *KMT2D*, *PIK3C2B*, and *TGFBR2*. The mean tumor mutational burden was 12.96 Mut/Mb (range, 2–36.05). The prevalence of other recurrent mutations, respectively, was less than 20% in the full TCGA cohort: NF1 (2.8%), KMT2D (16.2%), PIK3C2B (1.4%), and TGFBR2 (4.7%). Mutations in KMT2D and PIK3C2B were also associated with trends toward worse overall survival in TCGA HNSCC patients compared to WT (KMT2D, *p* = 0.10, PIK3C2B, *p* = 0.08), but mutations in the NF1 and TGFBR2 were not significantly associated with prognosis in TCGA (*p* = 0.8 for both, **Supplementary Figure S3**).

In HPV-positive tumors from the TCGA cohort, mutations were not found in NF1, PIK3C2B, or TGFBR2, and KMT2D mutations were seen in 13.3% of tumors. The frequency of KMT2D mutations in TCGA was not significantly different from the treatment-resistant cohort (13.3% vs. 40%, *p* = 0.12). Among HPV-positive tumors, KMT2D mutations were not associated with overall survival (*p* = 0.6, **Supplementary Figure S3**).

### Gene expression analysis

**Figure 2:**
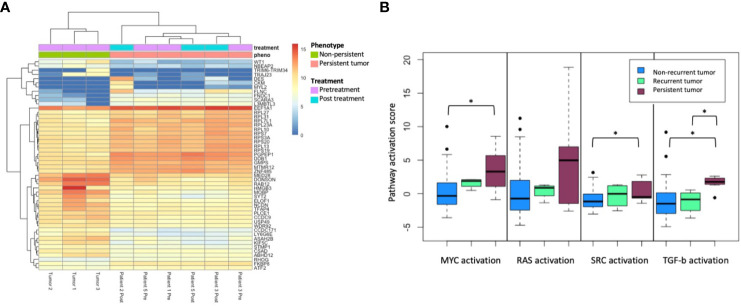
**(A)** Top 50 differentially expressed genes’ hierarchical clustering from between persistent tumors (red) and non-persistent tumors (green), FFPE only. **(B)** Pathway activation score for MYC, RAS, SRC, and TGF-b activation between non-recurrent tumors (n = 43), recurrent tumors (n = 4), and persistent tumors (n = 5). The asterisk (*) denotes p < 0.05 on paired t-test.

Evaluation of gene expression through RNA sequencing revealed alterations in specific pathways enriched in persistent tumors. Differential gene expression analysis was performed between persistent and non-persistent tumors (**Figure 2A**). The initial analysis was performed only in FFPE specimens (three non-persistent and four persistent tumors). Gene set enrichment analysis (GSEA) of hallmark pathways demonstrated a significant differential expression in the following major pathways: hypoxia (padj = 0.013), MYC targets (padj = 0.013), epithelial–mesenchymal transition (padj = 0.013), TGF-beta signaling (padj = 0.026), DNA Repair (padj = 0.026), interferon alpha response (padj = 0.026), IL2 STAT5 signaling (padj = 0.038), and p53 pathway (padj = 0.039).

Persistent tumors were then compared to non-recurrent HPV tumors from the previously published cohort, including 47 treatment-responsive tumors, of which four patients experienced a subsequent recurrence. Pathway activation analysis was performed in three groups: HPV-positive treatment-responsive tumors without recurrence (*n* = 43), treatment-responsive tumors with recurrence (*n* = 4), and persistent tumors (*n* = 4). Using DART pathway activation scoring and previously published pathways, gene expression profiling demonstrated a significant activation of MYC, SRC, and TGF-beta signaling pathways in persistent tumors compared to tumors with good prognoses, without recurrence (**Figure 2B**). Tumors with response to treatment but with a subsequent recurrence trended toward the intermediate activation of the MYC, SRC, RAS, and TGF-beta signaling pathways.

To understand the potential role of immune infiltrates, gene signature-based immunoprofiling was inferred from bulk sequencing. PD1 and PDL1 expression did not significantly differ in the persistent tumor cohort (*p* > 0.3). Overall immune score, stroma score, and microenvironment scores did not differ in the persistent tumor cohorts (*p* > 0.1). After multiple testing correction, three of 64 cell types were noted to have a significantly altered enrichment in persistent tumors: basophils (padj = 0.01) and Th2 cells (padj = 0.024), and pro B-cells showed increased enrichment (padj = 0.006).

### OncoGPS analysis and shift of genomic markers during treatment

**Figure 3:**
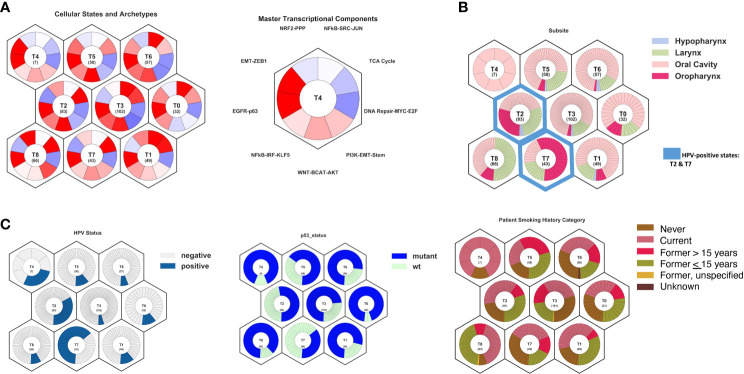
Model of head and neck squamous cell carcinoma tumor states and archetypes developed from The Caner Genome Atlas using Onco-GPS methods. **(A)** Summary of cellular states and archetypes, showing nine distinct cellular types, and transcriptional components used to build the cellular states, showing the key pathways to differentiate states. **(B)** Projection of tumor subsite onto cellular states, showing oropharyngeal tumors primarily in T2 and T7 states and larynx tumors in T8 state. **(C)** Projection of HPV status, TP53 mutation status, and smoking history onto cellular states, showing majority of the HPV-positive tumors in states T2 and T7. TP53 mutations were most dominant in states T0, T4, and T8. State T4 comprised primarily current smokers, and states T4, T5, and T8 had the fewest never smokers.

A model of HNSCC tumors was then defined using Onco-GPS methods (15) with 497 head and neck squamous cell carcinoma (HNSCC) tumors from The Cancer Genome Atlas (8). In order to test the hypothesis that persistent HPV-positive tumors match a phenotype that is more similar to HPV-negative disease, both HPV-positive and HPV-negative tumors were used to build a full Onco-GPS model. The tumors were defined by nine tumor states (**Figure 3A**), each characterized by the differential activity of nine key pathways: NRF2-PPP, NFkB-SRC-JUN, TCA Cycle, DNA Repair-MYC-E2F, PI3K-EMT-Stem, WNT-BCAT-AKT, NFkB-IRF-KLF5, EGFR-p63, and EMT-ZEB1. These pathways were selected using a cancer archetype methodology based on differential expression pathways that characterized head and neck cancers through GSEA analyses (**Supplementary Figure S1**). TCGA HNSCC tumors were then mapped to each subtype. HPV-positive and oropharynx tumors were clustered in states T2 and T7 (**Figures 3B, C**). Both states are characterized by the upregulation of EGFR-p63 and TCA Cycle pathways and the downregulation of EMT-ZEB1 and DNA Repair pathways. Interestingly, most laryngeal tumors were characterized as state T8 and additionally demonstrated NRF2-PPP and PI3K-EMT-Stem pathway activation. *TP53* mutations were most dominant in states T0, T4, and T8. State T4 was primarily comprised of current smokers, and states T4, T5, and T8 had the fewest never smokers.

**Figure 4:**
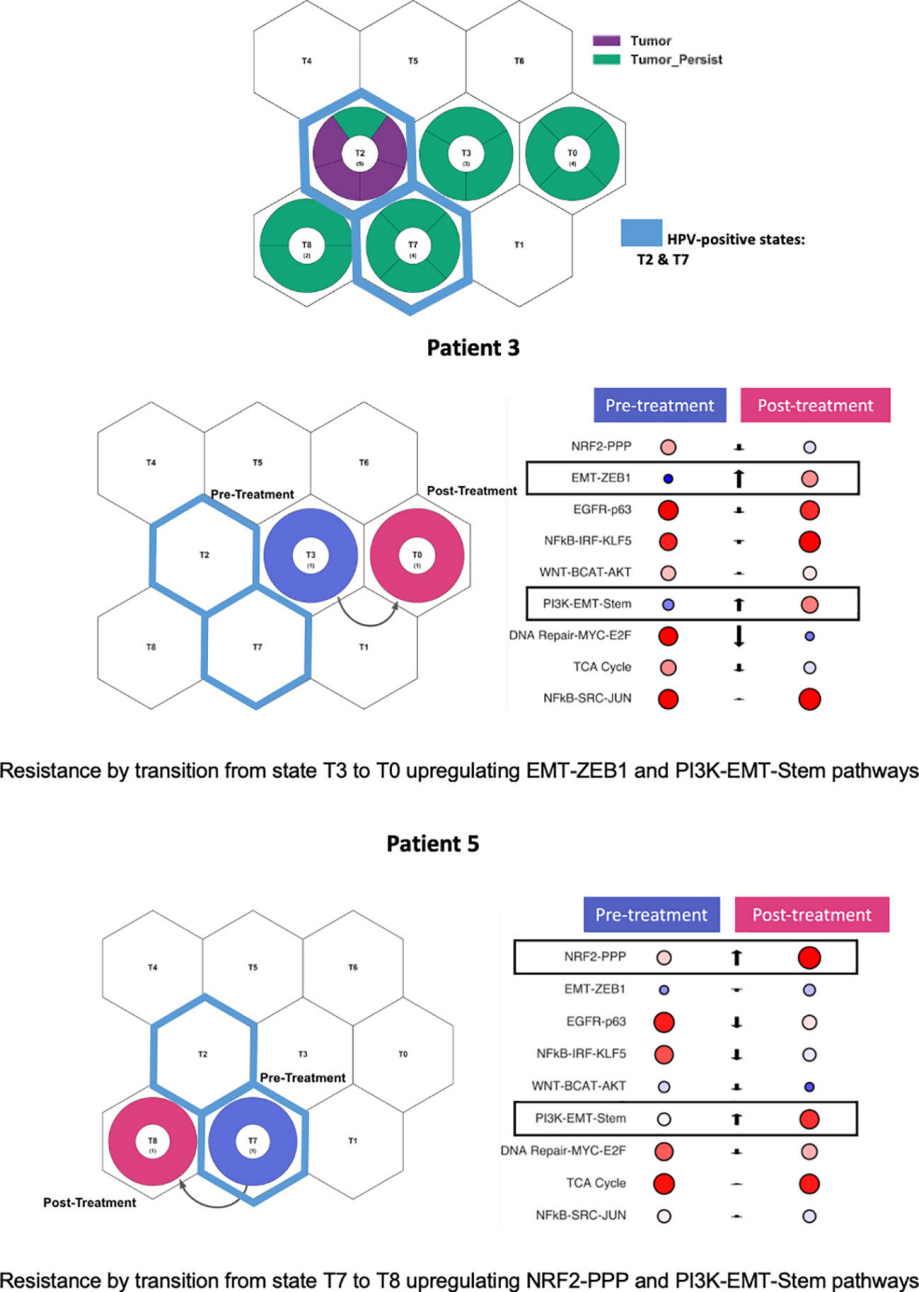
Persistent tumors projected onto tumor states and archetypes. Persistent tumors projected into both HPV-positive and HPV-negative states. Two patients with paired pretreatment and post-treatment samples. Patient 3 demonstrated cellular state shift from state T3 to T0. Patient 5 demonstrated cellular state shift from state T7 to T8. Both patients demonstrated upregulation of the PI3K-EMT-Stem pathway post-treatment.

When sequenced FFPE tumors were projected onto tumor states and archetypes, tumors responsive to treatment (purple) clustered in state T2, characterized by EGFR-p63/WNT and downregulation of EMT-ZEB1 and DNA Repair-MYC-E2F, and associated with HPV-positive oropharyngeal tumors (**Figure 4**). Two persistent tumors (green) were defined by T7 state (upregulation EGFR-p63 and TCA Cycle pathways and the downregulation of EMT-ZEB1 and DNA Repair pathways), an oropharyngeal/HPV-positive state. However, several persistent tumors were characterized by HPV-negative non-oropharyngeal states, including T0 and T3 states (predominantly the oral cavity, characterized by the upregulation of EGFR-p63 and NFkB-SRC-JUN pathways and the downregulation of DNA Repair-MYC-E2F) and T8 (predominantly larynx cancer and current/former smokers, characterized by the upregulation of NRF2-PPP and PI3K-EMT pathways).

Two patients (#3 and #5) had both pre- and post-treatment paired tumors for which RNA sequencing was performed. These paired tumor samples were projected onto tumor states. Both patients had tumors characterized by p53 mutations and had a prior smoking history. Pre- and post-treatment states and state pathway activation were evaluated for those patients (**Figure 4**). Patient #3 initially presented in state T3, shifting to T0 after treatment, characterized by the upregulation of the EMT-ZEB1 and PI3K-EMT-Stem pathways. Patient #5 initially presented in the HPV-positive T7 state but, during treatment, shifted to a post-treatment state dominated by larynx/smoking SCC type T8, with a significant upregulation of the NRF2-PPP and PI3K-EMT-Stem pathways. Thus, both persistent tumors demonstrated an upregulation of the PI3K-EMT pathway after platinum-based chemoradiation treatment. These state shifts suggest that the inhibition of PI3K and EMT pathways represent an opportunity to target treatment resistance in these tumors.

## Discussion

In the past two decades, our understanding of the impact of HPV-positive tumor status on clinical outcomes in oropharyngeal disease has expanded dramatically. The Ang et al. landmark study described the improved outcomes in HPV-positive disease (4), and we have continued to see an increasing incidence of HPV-positive oropharyngeal SCC (1). While most of these HPV-positive patients have an excellent response to therapy and remain disease-free, this disease continues to have a spectrum of patients who harbor an aggressive disease despite favorable biology. Within the RTOG 0522 cohort, locoregional failure was 17.3% and distant metastatic disease was 6.5% at 3 years for HPV-positive patients. Herein we described an even rarer phenomenon of patients who present with the persistent disease despite treatment with curative intent, which may occur in up to 5% of patients (3, 4).

Given this relatively infrequent disease phenotype, we were motivated to better understand the clinical characteristic as well as the genomic features of a treatment-resistant HPV-positive oropharyngeal disease. Within this rare clinical entity, we report a case series of patients and include the genomic analysis of this disease phenotype that has not previously been published. Notably, this patient cohort with treatment resistance included 30% never smokers. The patient age range was wide (43 to 87 years at the time of diagnosis), spanning the range of typical ages for the diagnosis of OPSCC. Most notably, the poor overall survival is highlighted in treatment-resistant diseases, with a median overall survival of 12.4 months. These patients have significantly worse prognosis compared to most HPV-positive patients with a 3-year OS of 82.4% (4) and even compared to HPV-positive patients who present with a recurrent disease, whose median OS after progression is 2.6 years (3). Treatment with salvage therapy was associated with some benefits, particularly salvage surgery, increasing the median OS to 21 months.

Mutational analysis in this cohort of treatment-resistant HPV-positive tumors demonstrated a much higher rate (60%) of p53 mutation compared to other published rates in the literature. In our previously published cohort, the p53 mutation rate was 2% in HPV-positive tumors (18), and the TCGA cohort demonstrated a similar rate of 2.2% (8). Another report detected up to 6.7% rate of p53 mutations in HPV-positive oropharyngeal cancer in a Japanese population (19). *TP53* mutations are a well-established risk factor for worse overall survival in HNSCC (20). Still its impact in the setting of HPV-positive diseases is not well described as these mutations rarely co-exist with HPV-mediated diseases. One study evaluating recurrent HPV-positive tumors demonstrated higher rates of p53 alterations in recurrent HPV-positive tumors (21). In the TCGA cohort, one HPV-positive patient demonstrated p53 mutation, and the overall survival was 12 months, similar to the median survival of the treatment-resistant cohort. Our data suggest that p53 mutation in the setting of HPV-positive OPSCC may portend a worse prognosis and may be a predictor of chemoradiation resistance. This may represent one biomarker to select patients for primary surgical therapy if treatment resistance is predicted.

The *TP53* mutations seen in the persistent disease cohort were observed in patients with either current or former smoking history. These mutations could contribute to the worse prognosis seen in HPV-positive smokers (4). Among smokers, indeed p53 mutations were associated with worse prognosis. However, given the rarity of these mutations in HPV-positive patients, conclusions still remain limited regarding the interplay between p53 and smoking in HPV-positive diseases. Additional investigation will also be needed to understand the mechanisms of disease in non-smokers who harbor an aggressive HPV-positive disease.

Previously published studies of mutational alterations in HPV-positive diseases have described other biomarkers associated with prognosis—for example, the loss of TRAF3 and PI3K pathway alterations has been associated with improved prognosis in HPV-positive tumors, including in a study of distant metastatic lesions (22, 23). *PIK3C2B* mutation was present in two patients in our cohort. Mutations were identified in KMT2D and PIK3C2B within the treatment-resistant cohort, and mutations in these genes demonstrated a trend toward worse overall survival in the TCGA cohort, suggesting possible biomarkers for aggressive diseases.

The genomic analysis demonstrated several key pathways activated in treatment-resistant HPV-positive tumors. GSEA identified MYC, EMT, TGF-beta, and DNA Repair as major pathways of differentially expressed genes compared to treatment-responsive tumors. DNA damage repair pathway enrichment may be associated with p53 mutation and loss. ERCC1 overexpression has also been associated with worse prognosis and treatment resistance in HNSCC (24, 25). Similarly, Hanna et al. described a trend toward greater alterations in DNA Repair proteins in distant metastatic HPV-positive diseases (23). Resistant tumors were also characterized by MYC, SRC, and TGF-beta pathway activation. HPV viral integration has been associated with MYC activation in multiple cancer types (26, 27); however, there have been mixed data on the prognostic significance of HPV integration (28–30). TGF-beta activation may be one mechanism of epithelial-to-mesenchymal transition (EMT), which has been related to treatment resistance with cetuximab (31), recurrent disease (32), as well as poor prognosis (33). Lastly, gene expression data was also utilized to explore differences in immune infiltrates and landscapes, but no significant differences were seen in PD1 expression, PDL1 expression, overall lymphocyte infiltrates, or immune scores. Only one patient demonstrated TMB greater than 10 mut/Mb, who may have derived benefit from immunotherapy (34).

Defining tumor states through OncoGPS archetypes allowed for the distilled characterization of the key pathway activation within each tumor. This demonstrated that treatment-resistant tumors primarily matched the cellular states of non-oropharyngeal HPV-negative tumors. Furthermore, both patients with pre- and post-treatment biopsy samples developed activation of the PI3K-EMT-Stem pathway during treatment. The activation of these pathways may represent an attractive potential target for salvage treatment in the setting of persistent disease after definitive chemoradiation with platinum therapy. Several PI3K pathway inhibitors, particularly mTOR inhibitors, have already been evaluated in clinical trials for HNSCC. While monotherapy with rapamycin and everolimus had limited clinical effect (35–37), dual targets with other inhibitors such as HER3 and MEK may offer more promise for these HPV-positive tumors characterized by PI3K activation (38–40).

This study is limited in scope due to the small patient cohort, limiting specific conclusions regarding both clinical behavior and genetic biologic alterations that may drive treatment-resistant HPV-positive oropharyngeal cancer. Specifically, in the genetic analysis, only half of the patient cohort and available tissue for sequencing analysis and sequencing were performed in archival FFPE samples, which can limit the quality. Tissue was collected when clinically available, resulting in some inconsistency in the use of pretreatment and post-treatment tissue. Specifically for those with clinical genomic testing, DNA sequencing was performed on both pretreatment and post-treatment tissue based on availability and clinician practice. Therefore, the identified alterations in post-treatment tissue could be acquired mutations that would not be as comparable to pretreatment tissue that was collected in the control cohorts. Furthermore, additional tissue validation such as with Sanger sequencing was unfortunately not feasible due to limited tissue availability from archival biopsy tissue. In addition, only two patients had tissue from both pre- and post-treatment available for comparison of change in response to treatment. Therefore, this represents a limited case series for which only preliminary conclusions can be drawn.

To improve the statistical power, treatment-resistant tumors were compared to previously published cohorts, including HPV-positive oropharyngeal cancer (7) and TCGA (8). Comparisons across cohorts can be confounded by significant batch effects. We sought to mitigate this by performing batch correction when performing gene expression analysis with treatment-responsive tumors in the previously published HPV-positive cohort, including three matched samples across cohorts (11). When using TCGA data, we utilized cellular archetype projections to mitigate batch effects when estimating pathway activation.

However, to our knowledge, this is the first published cohort to characterize this unique treatment-resistant phenotype in HPV-positive oropharyngeal disease. While a larger cohort is needed to provide additional validation, this study offers new insight into this rare disease phenotype.

## Conclusions

Chemoradiation-resistant HPV-positive OPSCC occurs infrequently but portends a dire prognosis compared to the majority of HPV-positive OPSCC. Specifically, patients with a persistent disease after treatment demonstrate a significantly worse prognosis; however, treatment with salvage surgery or systemic therapy can still increase the overall survival. These tumors demonstrate higher rates of *TP53* mutation, and *TP53* mutation may represent a biomarker for treatment resistance in HPV-positive OPSCC. The gene expression analysis also demonstrates that these resistant tumors demonstrate activation of the MYC, SRC, and TGF-beta pathways. Pathway mapping can further identify specific targeted therapies that may have higher efficacy than traditional platinum-based chemoradiation for tumors with this uniquely aggressive phenotype, such as PI3K pathway inhibition which is under development for the treatment of head and neck cancer.

## Data availability statement

The original contributions presented in the study are publicly available. This data can be found here: GEO repository, accession number GSE256047. The datasets presented in this study can also be found in the **Supplementary Material**.

## Ethics statement

The studies involving humans were approved by Johns Hopkins Institutional Review Board. The studies were conducted in accordance with the local legislation and institutional requirements. The ethics committee/institutional review board waived the requirement of written informed consent for participation from the participants or the participants’ legal guardians/next of kin because this is a retrospective review with no more than minimal risk.

## Author contributions

TG: Conceptualization, Data curation, Formal analysis, Investigation, Methodology, Visualization, Writing – original draft, Writing – review & editing. FZ: Data curation, Methodology, Writing – review & editing. ST: Data curation, Formal analysis, Methodology, Writing – review & editing. LC: Data curation, Formal analysis, Methodology, Writing – review & editing. LR: Data curation, Methodology, Writing – review & editing. PT: Formal analysis, Methodology, Visualization, Writing – review & editing. CF: Data curation, Methodology, Resources, Writing – review & editing. DG: Conceptualization, Project administration, Resources, Supervision, Writing – original draft, Writing – review & editing. RM: Conceptualization, Formal analysis, Funding acquisition, Resources, Supervision, Writing – original draft, Writing – review & editing.

## Supplementary material

Supplementary Figure 1 Defining tumor cellular states and archetypes in head and neck squamous cell carcinoma.

Supplementary Figure 2 Overall survival in months by p53 mutation status in The Caner Genome Atlas (TCGA) head and neck squamous cell carcinoma. **(A)** All TCGA patients [WT n = 144, mutant n = 362]. (B) TCGA HPV-positive patients [WT n = 44, mutant n = 1]. **(C)** TCGA current and former smokers [WT n = 99, mutant n = 275]. (D) TCGA never smokers [WT n = 41, mutant n = 78].
