# Supplementary figures and images for: Clinical and genomic characterization of chemoradiation-resistant HPV-positive oropharyngeal squamous cell carcinoma
Source: Front Oncol. 2024 Mar 5;14:1336577. doi: 10.3389/fonc.2024.1336577 (PMC10949886; doi:10.3389/fonc.2024.1336577)

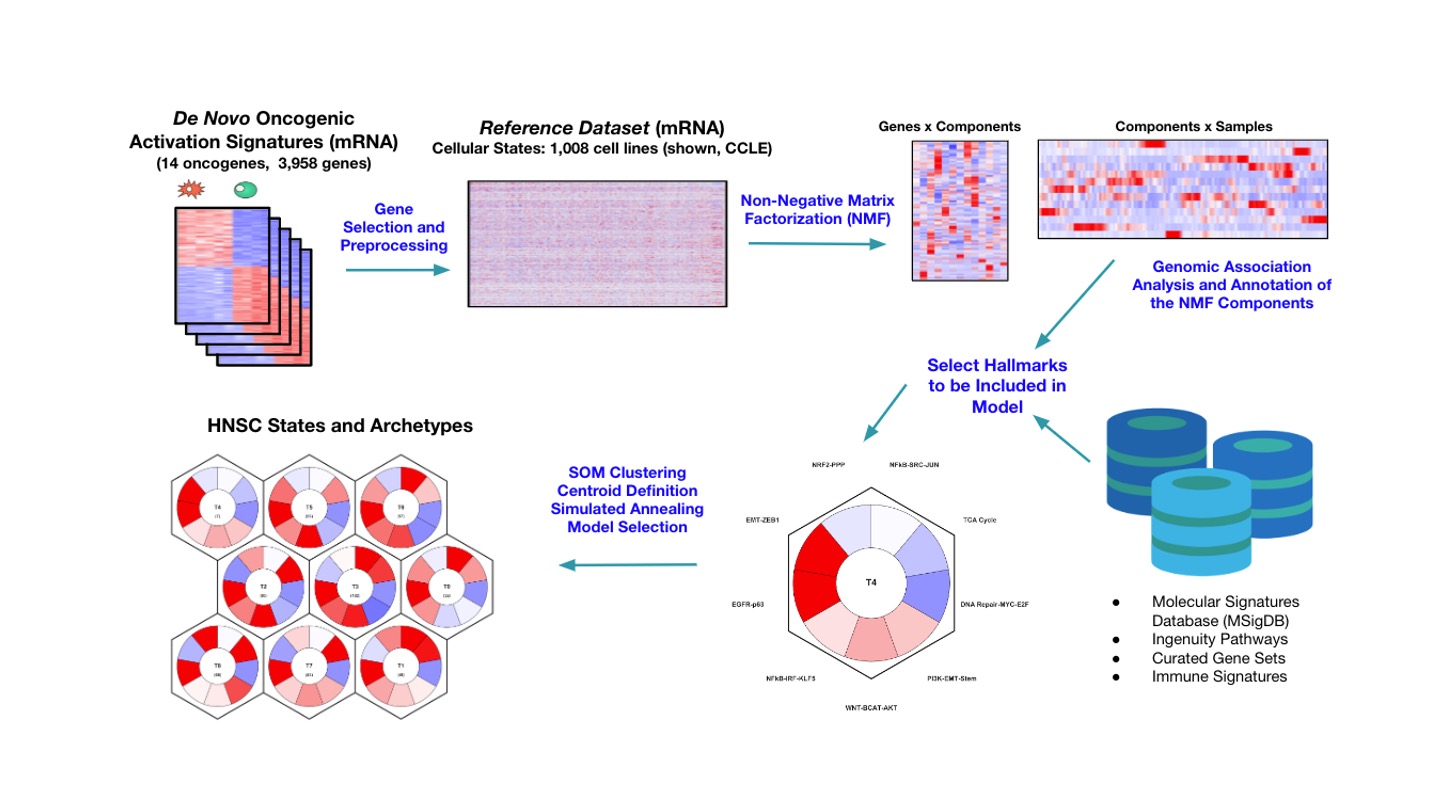

Supplement: Supplementary Figure 1 — Defining tumor cellular states and archetypes in head and neck squamous cell carcinoma. [file Image_1.jpeg]

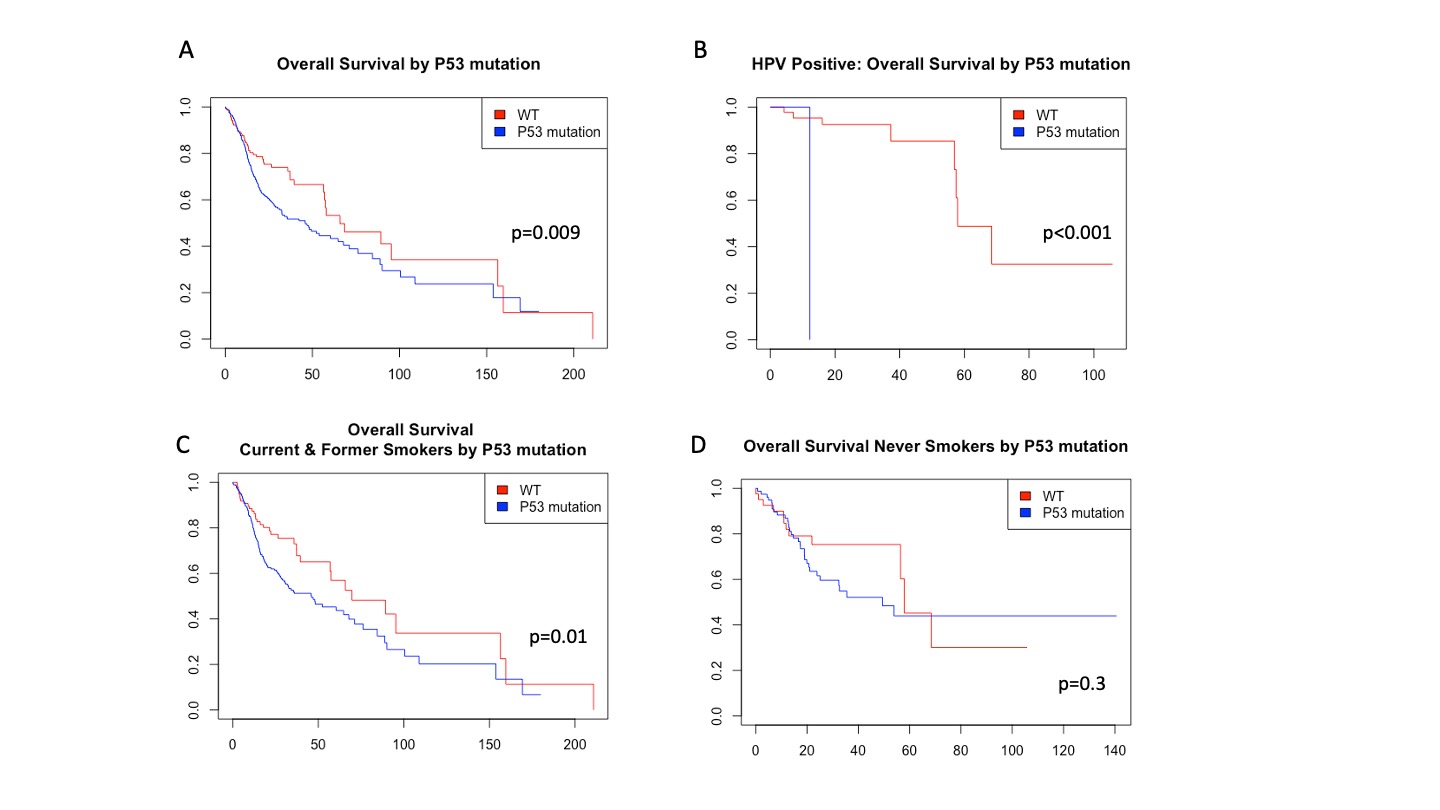

Supplement: Supplementary Figure 2 — Overall survival in months by p53 mutation status in The Caner Genome Atlas (TCGA) head and neck squamous cell carcinoma. (A) All TCGA patients [WT n = 144, mutant n = 362]. (B) TCGA HPV-positive patients [WT n = 44, mutant n = 1]. (C) TCGA current and former smokers [WT n = 99, mutant n = 275]. (D) TCGA never smokers [WT n = 41, mutant n = 78]. [file Image_2.jpeg]

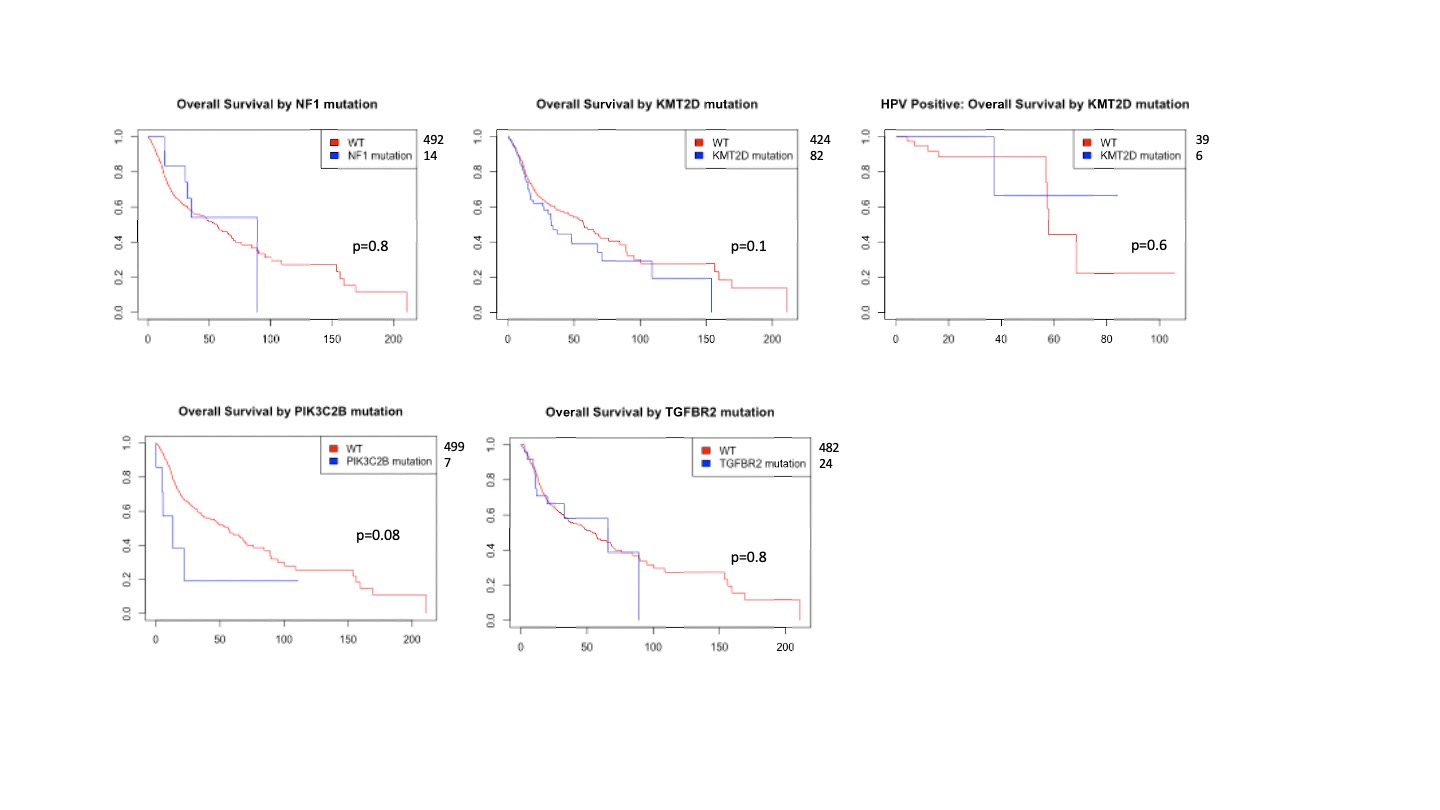

Supplement: Supplementary file 3 [file Image_3.jpeg]
